# A pilot study on sports activities in pediatric palliative care: just do it

**DOI:** 10.1186/s12904-023-01164-x

**Published:** 2023-04-19

**Authors:** Irene Avagnina, Anna Santini, Irene Maghini, Eleonora Salamon, Simonetta Papa, Luca Giacomelli, Giorgio Perilongo, Caterina Agosto, Franca Benini

**Affiliations:** 1 Paediatric Palliative Care, Pain Service, Department of Women’s and Children’s Health, University of Padua, Padua, 35127 Italy; 2 Polistudium SRL, Milan, Italy; 3 Pediatric Neurology and Neurophysiology Unit, Department of Women’s and Children’s Health, University of Padua, Padua, Italy

**Keywords:** Pediatric palliative care, Sport, Physical activity, Quality of life

## Abstract

**Background:**

There has been a growing interest in studying the value of physical exercise in children with disabilities or chronic health conditions because of evidence of improvement in quality of life, social acceptance, and physical functioning. However, only scant evidence exists for routine sports activities in children requiring pediatric palliative care (PPC), and in most cases, such evidence has been collected in oncological patients. The Pediatric Hospice of Padua is the referral center for PPC in the Veneto region (northern Italy). Starting from the experience of this PPC center, this pilot study aims to describe the personal experience of children and young people who practice physical activity and their caregivers’ perspectives, focusing particularly on the emotional and social impact of exercise and sports practice.

**Methods:**

Patients involved in at least one regular and structured sports activity were included in the pilot analysis. Two different ICF-CY (International Classification of Functioning, Disability and Health-Children and Youth Version) scales (“Body Function” and “Activity and Participation”) were filled out to assess the children’s global functional competence. Two online ad hoc questionnaires were created and administered to children, when able to respond, and caregivers.

**Results:**

A total of 9% of patients reported being involved in a sports activity. All children who played sports did not have indications of cognitive retardation. The most practiced sport was swimming. The use of standardized methods such as ICF-CY shown that severe motor impairments do not limit sports accessibility. Questionnaires result suggest that sports are a positive experience for both children needing PPC and their parents. Children encourage other children to do sports and can find the positive side even in difficulties.

**Conclusion:**

Since PPC is encouraged as early as the diagnosis of incurable pathologies, the inclusion of sports activities in the context of a PPC plan should be considered from the perspective of improving quality of life.

**Supplementary Information:**

The online version contains supplementary material available at 10.1186/s12904-023-01164-x.

## Background

WHO defines pediatric palliative care (PPC) as ‘*the active total care of the child’s body, mind, and spirit that also involves giving support to the family’* [1]. According to the recent standards in the field [2], PPC should be instituted as early as possible – when the illness is diagnosed or even earlier – and should be continued regardless of whether a child receives treatment for the disease. Thanks to progress in PPC knowledge and the increasing availability of advanced medical devices, PPC can be continued for years. Therefore, an adequate PPC plan should promote cognitive, emotional and social development and physical growth. It also should enable recovery, support, and strengthening of motor, communication, sensory, cognitive, and relational functions [2].

There is consensus on the positive impact of participation in sports activities on children with disabilities in terms of quality of life, social acceptance, and physical functioning [3–5]. Additionally, WHO has recently stressed this point in the guidelines on physical activity and sedentary behavior issued in 2020 [6]. Accordingly, some studies have described and analyzed the characteristics and participation in physical and sports activities of children with chronic health conditions, disability, and/or atypical development, as well as the psycho-physical well-being factors involved [7–11]. Studies on evaluating barriers and facilitators of sports activities for disabilities are also available [7, 12, 13]. Otherwise, there is a shortage of studies specifically addressing the sports experience of patients needing PPC services and their caregivers. As a result, gaps still remain in the understanding of how practicing sports benefits the emotional and social spheres.

Starting from the experience of our PPC referral center, the aim of this pilot study was to describe the personal experience of children and young people who practice physical activity and their caregivers’ perspectives, focusing particularly on the emotional and social impact of exercise and sports practice.

## Methods

### Study design

This pilot study was conducted at the Pediatric Palliative Care Center of Padua (Padua, Italy) in a pure clinical practice setting to describe the sport experience and assess the emotional and social impact of exercise and sports practice in pediatric patients and their caregivers. A functional assessment tool was used by physicians to assess the global functioning of children practicing sports and two ad hoc questionnaires were given to children and caregivers to assess their personal experience on sports (see the following paragraphs for details). The clinical charts of all included patients were retrospectively analyzed to retrieve demographic and clinical information.

### Setting

The Pediatric Hospice of Padua is the referral center for PPC in the Veneto region (northern Italy). It provides care for approximately 250 patients a year, aged 0–23 years, with different incurable disease (oncological and non-oncological), offering both in hospice and home assistance and coordinating the healthcare network involved in the care of the child.

### Population

All patients cared for at the Pediatric Palliative Care center of Padua in March 2020 were considered for the analysis. Patients involved in at least one regular and structured sports activity were included. One caregiver for each patient practicing sport was also considered in the study.

### Global functional competence

The ICF-CY (International Classification of Functioning, Disability and Health-Children and Youth Version) is a conceptual model for describing health, functioning, and disability, which assesses the ability to perform activities and complete bodily functions from a predefined list. It represents a standardized tool for the evaluation of child’s function. The assessment is given on a 5-point scale, where 0 means no impairment and 4 means total disability [14, 15]. The treating physicians filled out two different ICF-CY scales (“Body Function” and “Activity and Participation”) to assess the children’s global functional competence.

### Questionnaires

Two online ad hoc questionnaires were created and administered to children, when able to respond, and caregivers (only one caregiver was asked to complete the questionnaire). These questionnaires were aimed at providing the child’s and caregiver’s perspectives on the importance of physical activity (Annex I and Annex II).

The caregiver questionnaire investigated demographic data (11 items), characteristics of the sport practiced by the child (12 items), sports experience (four items), caregiving stress (one item), economic issues (one item), personal satisfaction (three items), skills and achievements (seven items), personal experience with sports (three items), personal evaluation on the child’s perspective (one item, multiple-choice responses), facilitators (six items) and barriers to sports (seven items). Two open-ended questions about the general vision of the sports experience and the perceived consequences for their own lives were also included. All items were assessed through a 5-point Likert scale, except for demographic data and sports characteristics.

The children questionnaire investigated demographic data (five items), sports experience (1 item), stress related to sports activities (one item), skills and achievements (seven items), and personal sports experiences (six items). Three open-ended questions about the perceived consequences for their own lives, the general vision of the experience, and the perspectives on the future were also included. All items were assessed through a 4-point scale, except for demographic data. In the pediatric questionnaire, we decided to use a 4-point scale to facilitate the participation of children of different ages.

The compilation of the questionnaires took about 30 min. The questionnaires were delivered separately to the children and the caregiver to allow everyone to express themselves freely.

### Data collection and data analysis

Data were collected in complete anonymity through the delivery of a paper copy of the questionnaire.

For quantitative analysis, data were analyzed through descriptive statistics. All data were analyzed on the entire population.

For the qualitative analysis, the open-ended questions were divided by thematic analysis to identify some macro response areas [16, 17]. This process was carried out according to the indications of Nowel and collaborators; an inductive process was used, and the categories were defined based on the principles of grounded theory [17, 18].

### Ethical considerations

The study was conducted in accordance with the ethical standards as laid down in the 1964 Declaration of Helsinki and its later amendments and within the protocol notified to the ethics committee of the Hospital University of Padua (protocol number 0071163). Verbal informed consent to participate was obtained from the parents or legal guardians of any participant under the age of 18. Verbal informed consent to participate was obtained from all other participants above the age of 18, i.e., caregivers.

## Results

### Patients

Considering both hospice and home care, a total of 177 patients were followed by our PPC center in March 2020 and all of them were screened. Of them, 16 (9%) were involved in at least one regular, structured sports activity. All of them participated in the questionnaire, along with one caregiver for each one.

Their baseline characteristics are depicted in Table 1. Nine patients (56%) were affected from neuromuscular disease (Spinal Muscular Atrophy, SMA 2, n = 5; SMA 3, n = 4). Most patients were receiving professional physiotherapy (n = 11, 69%), in most cases for 1 h per week. Half of the patients (n = 8, 50%) were in a wheelchair. From a socioeconomic perspective, most families of enrolled children had a low-mid total income (< 30,000€/year; n = 13, 81%), and all were receiving state economic benefits.

**Table 1:** Baseline characteristics of the children. Please format tables like the submitted version, there are lots of inconsistencies with the tables, sub headings, etc

Demography	N = 16
Sex	
Male{THESE NEED TO GO UNDER SEX AND HAVE BULLET POINTS} Female	10 (62.5%) 6 (37.5%)
Age (years)	
Mean (SD), min–max	12.3 (± 3.3), 6–18
Diagnosis Neuromuscular disease{ADD BULLET POINTS TO THIS}	9 (56.3%)
Myopathy	4 (25%)
Charge syndrome	1 (6.3%)
Central hypoventilation syndrome	2 (12.5%)
Disability Physical	15 (93.7%)
Both physical and visual	1 (6.3%)
School Kindergarten{ADD BULLET POINTS TO THIS} Primary school{ADD BULLET POINTS TO THIS} Lower secondary school{ADD BULLET POINTS TO THIS} Upper secondary school{ADD BULLET POINTS TO THIS}	1 (6.3%) 4 (25%) 7 (43.8%) 4 (25%)
**Therapy**	**N = 16**
Physiokinesitherapy	11 (68.8%)
1 h/week	6 (54.5%)
2 h/week	3 (27.3%)
3 h/week	2 (18.2%)
**Aids and orthoses**	**N = 16**
Wheelchair	8 (50%)
Wheelchair and braces	3 (18.8%)
Wheelchair and braces and augmentative communication aids	1 (6.3%)
Wheelchair and braces and corset	1 (6.3%)
Wheelchair and braces and corset and walker	1 (6.3%)
Wheelchair and other	1 (6.3%)
None	1 (6.3%)

### Global functional competence

The evaluation of the two areas of the ICF-CY showed that children who played sports mainly had a problem with fatigue, intended as impaired exercise tolerance (n = 11, 71%) and muscle strength (n = 6, 37%). Most children had no pain or suffering (n = 14, 87%) nor alterations of emotional function (n = 13, 81%).

Regarding the daily activities, 76% (n = 12) of participants did not walk, 56% (n = 9) were not able to move independently, 50% (n = 8) were not independent for personal hygiene, and 63% (n = 10) were not able to dress by themselves. All the participants were able to move independently with the equipment. Almost all participants (n = 15, 94%) had no entertainment problems, 87% (n = 14) had no problems in social interactions, and 69% (n = 11) had no problems managing physical and psychological stress.

### Sports activities

Sports activities played by the patients are summarized in Table 2. Swimming was the most frequently practiced sport (n = 7, 44%), followed by wheelchair hockey and horse riding (n = 3, 19% each). The mean age at the initiation of sports activity was 12.3 years (range, 6–18). In most cases, children attended their sports class once weekly (n = 10, 62%). A total of five children participated in some competitions every month (n = 5, 44%). The wide majority of children participated in team activities (n = 13, 82%).

**Table 2:** Sports activities played by the patients. (source: original)

Played sport	n = 16, n (%)
Swimming	4 (25.0%)
Swimming and wheelchair basketball	1 (6.3%)
Swimming and horse riding	1 (6.3%)
Swimming and fencing	1 (6.3%)
Wheelchair basketball	1 (6.3%)
Football	1 (6.3%)
Dance	1 (6.3%)
Horse riding	2 (12.5%)
Wheelchair hockey	3 (18.8%)
Motor activity	1 (6.3%)
**Age at the start of sporting activities (years)**	7.7 (± 3.9), 1–15 N
**Number of sports classes/weeks**	
One	10 (62.5%)
Two	5 (31.3%)
Three or more	1 (6.3%)
**Participation in a team activity**	
Always	7 (43.8%)
Often	6 (37.5%)
Sometimes	1 (6.3%)
Never	2 (12.5%)
**Possibility of playing sports**	
Sports society	7 (43.8%)
Association	6 (37.5%)
Gym	1 (6.3%)
Other	2 (12.5%)

According to children, 14 of them (87%) decided on their own to play sports; on the other hand, according to caregivers, six children (37%) decided on their own to play sports, in nine cases (56%) children were introduced to sports by their caregivers and one family (6%) followed the socio-health professionals’ indication.

Concerning sports, families learned about parasports through local sports clubs (n = 8, 50%), disease-specific organizations (n = 6, 38%), and word of mouth (n = 2, 12%). None found information through the national healthcare system.

Football was the most frequently followed sport (n = 9, 56%). Most children desired to play football (n = 11, 69%; eight males and three females), but only one actually played this sport. Other desired sports activities were swimming (n = 2, 12%; both patients were actually practicing swimming), dancing, basketball, and horse riding (n = 1 for all, 6%). Overall, five children (31%) played the sport they wished.

Sports class characteristics are reported in Table S1. Most classes were composed of a mixed group of adults and children (n = 13, 81%) and were specifically meant for people with disabilities (n = 12, 75%).

All children were driven to their sports class by their caregivers; most children did not require any specific equipment to practice sports (n = 9, 56%; Table S2), but for those who did, only one (6%) received financial support from the healthcare system.

### Perspectives on physical activity

#### Caregivers

The local experience with sports was generally considered very satisfactory and supported by trained coaches (n = 13, 81%). There was no caregiving stress; it was not considered a problem for families to organize themselves according to sports training (not at all/little, n = 12, 76%). Few families considered the required economic investment considerable (very much, n = 4, 25%). On the personal experience side, sports enabled parents to have a happier social life and generally improved the quality of life for half of the participants (n = 8, 50%). Half of the respondents reported that their child’s sport allowed them to establish important bonds with other parents (a lot, n = 8, 50%).

The thematic analysis of the open questions led to the identification of different thematic clusters (Tables S3 and S4). Regarding the perceived feelings watching the child play sports identified by the verbatim quotes, pride (n = 7, 44%), happiness (n = 7, 44%), satisfaction (n = 2; 12%), and equality to other parents (n = 1; 6%) emerged (Table S3). With regard to the suggestions for a family who is on the fence about having their child with a disability start a sports activity, encouragement (n = 10; 63%), socialization (n = 2; 12%), equality (n = 2; 12%) and physical and psychological well-being (n = 4; 24%) were the areas identified by the thematic analysis (Table S4).

#### Children

Most children found a comfortable situation and prepared coaches in their territory (n = 10, 63%). Half of the participants desired to carry out sports activities with peers without disabilities (n = 8, 50%), although some never thought about this opportunity (n = 4, 25%), and the other 25% did not mind playing sports with people with disability.

Six children (38%) reported that reconciling sports training with school commitments could be quite taxing.

The thematic analysis of the open questions led to the identification of different thematic clusters (Tables S5 and S6). In particular, children appreciated the social side of the sport (n = 7, 44%), the feeling of freedom linked to the sporting gesture (n = 7, 44%) and the competition (n = 1; 6%; Table S5). Most of the children were not afraid of not being able to play sports in the future (n = 12, 76%).

Children would invite other children with the same condition to play sports (encouragement; n = 10, 63%), also focusing on the positive value of sports on psycho-physical well-being (n = 6, 42%; Table S6).

### Comparison between caregivers’ and children’s perspectives on physical activity

As resumed in Table 3, caregivers thought that sports encouraged autonomy more than children (81% versus 69%). All caregivers were convinced that improved self-esteem and sociability were relevant aspects of practicing a sport. Conversely, children perceived that sport had minor positive effects on both self-esteem (75%) and socialization (56%). For caregivers, sports promoted learning rules and discipline (69%), while only 38% of children found this true. In addition, all caregivers saw sports as a driver for achieving personal goals, while only 69% of children agreed on this. For the latter, no new motor skills could be acquired through sports activity (38%), in disagreement with caregivers who felt so in the majority (81%).

**Table 3:** Children’s and caregivers’ responses to the ad hoc questionnaire. (source: original)

Statement	Respondent	Little	Sufficiently	Much
Comfortable situation and prepared friendly trainers	Caregivers	–	3 (19%)	13 (81%)
	Children	–	6 (38%)	10 (63%)
Taxing to organize according to training	Caregivers	12 (75%)	4 (25%)	–
	Children	2 (13%)	8 (50%)	6 (38%)
Fostering autonomy	Caregivers	1 (6%)	2 (13%)	13 (81%)
	Children	2 (13%)	3 (19%)	11 (69%)
Boosting self-esteem	Caregivers	–	–	16 (100%)
	Children	1 (6%)	3 (19%)	12 (75%)
Encouraging socialization	Caregivers	–	–	16 (100%)
	Children	–	6 (38%)	9 (56%)
Promoting rules and discipline	Caregivers	–	5 (31%)	11 (69%)
	Children	3 (19%)	6 (38%)	6 (38%)
Having and achieving goals	Caregivers	–	–	16 (100%)
	Children	1 (6%)	4 (25%)	11 (69%)
New motor skills	Caregivers	1 (6%)	2 (13%)	13 (81%)
	Children	2 (13%)	8 (50%)	6 (38%)
Allowing not perceiving disability as a limitation	Caregivers	1 (6%)	2 (13%)	13 (81%)
	Children	3 (19%)	4 (25%)	9 (56%)
Fear of increased health risks	Caregivers	14 (88%)	2 (13%)	–
	Children	13 (81%)	2 (13%)	1 (6%)
Improving psycho-physical health	Caregivers	–	1 (6%)	15 (94%)
	Children	–	2 (13%)	14 (88%)

Most caregivers (81%) felt that sports enabled reducing the perception of disability-related limitations, while only 56% of children had this perception. Instead, there was an agreement that sports would not increase health risks (88% parents, 81% children), that it was an aid to psycho-physical health (94% parents, 88% children), and, above all, that it was a fun activity (62% of parents, 81% of children).

### Barriers and facilitators

The accessibility to the structures in the territory (n = 9, 63%), the presence of competent operators (n = 13, 82%), the adaptation of environments (n = 9, 57%), and the presence of adequate communication supports (n = 9, 56%) were mainly mentioned by parents as elements capable of facilitating sports activities in disability contexts.

The major barriers perceived by caregivers were mostly those of the environment, unsuitable spaces (n = 8, 50%), and architectural barriers (n = 10, 62%). Cultural barriers were also considered relevant (n = 9, 57%), as well as the lack of support outside the family (n = 8, 50%; Table S7).

## Discussion

There has been a growing interest in studying the value of physical exercise in children with disabilities or chronic health conditions because of evidence of improvement in quality of life, social acceptance, and physical functioning [7–11, 19, 20]. However, only scant evidence exists for routine sports activities in children requiring PPC, and in most cases, such evidence has been collected in oncological patients; thus, evidence on the impact of physical activity in children with other diagnoses is lacking [21, 22]. Furthermore, although it is widely accepted that the benefits of exercise and sports practice go far beyond physical function endpoints, gaps still remain in understanding how they benefit the emotional and social spheres.

To the best of our knowledge, our study is the first carried out in a PPC referral center describing sport activities and reporting the emotional and social impact of sports practice not only for the caregiver but also for patients. In our center, 9% of patients reported being involved in a sports activity. Importantly, the eligibility characteristics of PPC include the presence of a life-threatening or life-limiting illness combined with the high complexity of care. In this context, children very often present with psychomotor retardation. In our cohort, all children who played sports did not have indications of cognitive retardation in their medical history. This seems to be a predominant criterion for the possibility of inclusion in sports activities.

The assessment of the global functional competence through the ICF-CY, a valuable tool for standardizing the child’s functional evaluation criteria, highlights how the presence of relevant motor difficulties does not limit the possibility of playing sports. A shared patient characteristic in the ICF-CY evaluation was the ability to use aids or equipment autonomously and, for most participants, to relate and interact with others. These aspects become more relevant in team sports, where team interaction is an integral part of the activity. Besides the need to promote sports inclusion even in patients with severe motor disabilities, using ICF-CY could be a useful tool to better target the type of sports to be suggested based on the residual autonomy and relationship skills.

Although sports equipment is often needed, the general economic situation of the family did not seem to be a limitation to sports practice; therefore, affordability of sports activities seemed to be guaranteed. Nevertheless, the major involvement of the healthcare system should be considered.

The most practiced sport was swimming, as children with disabilities easily do it. At the same time, it is the most recommended sport in the context of classic rehabilitation activities.

Another value of our study was the possibility of comparing the agreement between caregivers’ and children’s responses to the same question. Caregivers widely agreed on the view of sports as a self-esteem and socialization-stimulating activity that promotes respect for rules and achievement of goals. Otherwise, children showed a greater variability of responses in this regard. This can be because parents may have an idealized view of sports activity, while children tend to be more realistic and judge their experience in a personal way. At the same time, caregivers and children agreed on not being afraid of greater health risks and considering sports good for psycho-physical health. In evaluating their own experience, parents expressed pride, happiness, and satisfaction with watching their children playing sports, focusing their thoughts on emotions; conversely, children focused on the social impact of sports activities and their feeling of freedom, then reported their satisfaction with the activities.

Regarding facilitators and barriers to sports activity under conditions of disability, our findings align with the literature data [12, 13, 23, 24]. The most cited limits are related to physical environments, architectural barriers, and more generally cultural barriers. Otherwise, unlike the literature data, transportation is not perceived as a barrier to sports activity, perhaps because parents are used to being the primary caregiver and being autonomous in traveling with their children, which also occurs for rehabilitative therapies and hospital visits.

Our study has some limitations that need to be acknowledged, such as the small population involving mainly patients with neuromuscular pathology and the lack of analysis concerning the characteristics of caregivers. In addition, ICF-CY is designed for children, adolescents and young adults and some issues (such as sexual development or household tasks) are not assessable in the pediatric context. At the same time, our study represents the only description of the impact of sports activities on a children population needing PPC.

## Conclusion

Our findings suggest that sports are a positive experience for both children needing PPC and their parents. Children encourage other children to do sports and can find the positive side even in difficulties. The use of standardized methods, such as ICF-CY, has shown that severe motor impairments do not limit sports accessibility. Since PPC is encouraged as early as the diagnosis of incurable pathologies, the inclusion of sports activities in the context of a PPC plan should be considered from the perspective of improving quality of life.

## Electronic supplementary material

Below is the link to the electronic supplementary material.

Additional files: Annex I - Sports Questionnaire: Children and teens. Title of data: Sports Questionnaire ? Children and teens. Description of data: Questionnaire administered to children and teens.

Annex II - Sports Questionnaire ? Caregivers. Title of data: Sports Questionnaire: Caregivers. Description of data: Questionnaire administered to caregivers.

Supplementary material: Title of data: Supplementary Tables 1, 2, 3, 4, 5, 6. Description of data: Sports class characteristics, Equipment for sport, Barriers to sports activities for children in PPC, Thematic analyses.

## Data Availability

The datasets used and/or analyzed during the current study are available from the corresponding author on reasonable request.

## Abbreviations

PPC: Pediatric palliative care
ICF-CY: International Classification of Functioning, Disability and Health-Children and Youth Version
SMA: Spinal Muscular Atrophy
