# Supplementary material for: A pilot study on sports activities in pediatric palliative care: just do it
Source: BMC Palliat Care. 2023 Apr 19;22:45. doi: 10.1186/s12904-023-01164-x (PMC10114486; doi:10.1186/s12904-023-01164-x)
Supplement: Supplementary file 1 — Additional files: Annex I - Sports Questionnaire: Children and teens. Title of data: Sports Questionnaire ? Children and teens. Description of data: Questionnaire administered to children and teens. [file 12904_2023_1164_MOESM1_ESM.docx]

Sports Questionnaire - Children and Teens

Hello!

Thank you for taking the time to do this interview. We want to ask you some questions about sports to learn about your experience, what you like, and how you feel about playing sports.

It is important that you fill out the questionnaire when you are relaxed and in a place where no one will bother you. Remember: no answer is right or wrong; it is important for us that you are honest.

We ask very little of your time; you’ll see that in 15 minutes, you'll have everything filled out!

Please note that your questionnaire is saved with those of other children and teens, no one will know your identity, and all your answers will be used anonymously. No one will be able to trace your identity.

Thank you, enjoy the questionnaire!

*Required questions

1. Email *

The questions below are about the sport you play and what you think about it.

The questionnaire

1. What is your name? Please, write your name and surname *
2. Did you choose to play sports? *

*Mark only one oval.*

Yes

No

1. What is your favorite sport to watch? *
2. What sports do you practice now? *
3. What sport would you have liked to do most of all? *
4. How much do you agree with these statements: *

For each statement, choose how much you agree between "not at all," "fairly," and "very much." If you don't know what to answer, click on the "don't know" box.

*Mark only one oval per line.*

Not at all Fairly Very much Don’t know

I immediately found an accepting team where I felt at ease

It's easy to fit my school schedule with sports

I would like to do sports with people without disabilities

Sports made me feel that I can do things on my own.

Sports have improved my self-esteem

Thanks to sports, I have more friends

Sports have helped me respect the rules

Sports helped me give myself goals and achieve them

Sports have allowed me to make movements or have the strength I didn't have before.

Sports help me see disability not as a limitation

Sports mean fun to me

Sports are rehabilitation activities for me,

like physical therapy

Sports allow me to express myself

Sports make me feel good psychologically and physically

By playing sports, I am more afraid of getting sick or injured (e.g., fractures or falls)

1. What do you like most about playing sports? *
2. Are you afraid you won't be able to play sports in the future? Why? *
3. What would you say to a child with disabilities who is unsure whether to start a sports activity? *
