# Supplementary material for: A pilot study on sports activities in pediatric palliative care: just do it
Source: BMC Palliat Care. 2023 Apr 19;22:45. doi: 10.1186/s12904-023-01164-x (PMC10114486; doi:10.1186/s12904-023-01164-x)
Supplement: Supplementary file 2 — Annex II - Sports Questionnaire ? Caregivers. Title of data: Sports Questionnaire: Caregivers. Description of data: Questionnaire administered to caregivers. [file 12904_2023_1164_MOESM2_ESM.docx]

Sports Questionnaire – Caregiver

Dear participant,

Thank you for taking the time to fill out this interview.

We are conducting a survey within the Padua Pediatric Hospice to assess the personal experience of children/teens who practice sports and their caregivers.

You are the primary caregiver, the one who takes care of most of the time and needs of your child or children. We know how central and crucial your support is in enabling your son/daughter to achieve small and big goals.

It is important to fill out the questionnaire at a convenient time and in a quiet place. You will be asked personal and subjective questions. There are no right or wrong answers; we kindly ask you to respond honestly. The compilation takes about 15 minutes to complete.

We remind you that if you change your mind, you can decide to end the completion at any time by simply closing the window.

Please note that all data and information collected will be managed by the Research Promoter - UOSD Pain Therapy and Pediatric Palliative Care - Pediatric Hospice and will be only made accessible for research purposes anonymously. This ensures that your identity or that of your son/daughter cannot be traced.

In addition, all data will be processed according to the laws D.Lgs 196/2003 and UE GDPR 679/2016.

Thank you, enjoy the questionnaire!

*Required questions

Socio-personal data sheetThis series of questions is about your child and your family.

# Please provide your child's first and last name. *

We need this information to merge data from this questionnaire with clinical data. The data will then be used in aggregate and respecting the privacy of all participants. Therefore, it will not be possible to trace the participants’ identities.

# Please specify the sex of your child. *

## Mark only one oval.

Male Female

# Please indicate the age of your child. *

1. What is your child's type of disability? *

(You can select one or more answers)

*Select all applicable entries.*

Physical disability Mental disability

Visual impairment Hearing impairment

# Please indicate your child's diagnosis. *

1. What school does your child attend? *

## Mark only one oval.

Kindergarten Primary School

Middle School

High School

University

Not attending school

# What kind of rehabilitation therapy does your child do during the week? *

(You can select one or more answers)

*Select all applicable entries.*

Occupational therapy Physiotherapy

Speech therapy Neuropsychomotricity

No rehabilitation therapy

Other:

# How many hours of rehabilitation therapy does your child do in total in a week? *

## Mark only one oval.

One hour per week

Two hours per week

Two to four hours per week

More than four hours per week

My child does not do any rehabilitation therapy during the week

# What kind of aids and orthotics does your child use? a. wheelchair, b. walker, c. augmentative communication aids (e.g., tablet), d. lower or upper limb braces and orthopedic shoes, e. bust, f. other.

(You can select one or more answers)

*Select all applicable entries.*

Wheelchair

Walker

Augmentative communication aids

Lower or upper limb braces and orthopedic shoes
 Bust

My child does not use any aids or orthotics

Other:

# What is your household's approximate ISEE income bracket? *

## Mark only one oval.

Less than 10,000 euros Between 10,000 and 30,000 euros

Between 30,000 and 50,000 euros

Over 50,000 euros

# What government benefits do you receive? *

(You can select one or more answers)

*Select all applicable entries.*

None

Disability allowance ICD-a

ICD-b ICD-p ICD-m

Blindness allowance Project funds

Other:

Sports Questionnaire – Caregiver

These questions are specific to your and your child's experience with sports activities.

1. What kind of sports does your child practice? *

# How old was your child when they started playing sports? *

1. How many times a week does your child play sports (excluding friendly or competitive games)? *

## Mark only one oval.

Once a week
Twice a week

Three or more times a week

## How many gatherings with other teams (e.g., friendly or competitive games) or competitions are scheduled in an average month? *

## Mark only one oval.

No gatherings with other teams or competitions are scheduled

Once a month

Twice a month

Three or more times a month

# Was your child able to participate in team activities during the past year? *

## Mark only one oval.

Always

Many times

Sometimes Never

1. Where did you find opportunities for your child to play sports? *

## Mark only one oval.

Sports club Association

Gym

Other:

# Who advised you first to have your child play sports? *

## Mark only one oval.

My child asked for it

We, parents or family members

Social and healthcare professionals (pediatrician or general practitioner, rehabilitation services, social services, psychologist, school institution)

Other:

# Who does your child play sports with? *

## Mark only one oval.

Age peer group Adult group

Mixed group

# Does the group consist totally or partially of other people with disabilities? *

## Mark only one oval.

Yes

No

# Is the group composed of males, females, or is it a mixed group? *

## Mark only one oval.

Male group Female group

Mixed group

# Who takes your child to practice most of the time? *

## Mark only one oval.

We, parents

Association or sports club
Other:

# If your child requires wheelchairs or aids other than the usual ones, how did you get them?

## Mark only one oval.

My child does not need any aids to play sports Prescription charged to the National Health System
Cost-sharing

Purchase charged to the family

Other:

# How much do you agree with the following statements: *

*Mark only one oval per line.*

Not at all Little Fairly Much Very much

I immediately found a welcoming situation and trained coaches

My area offers few sports activities for children with disabilities

I believe that playing sports with people without disabilities can be a positive aspect for my child

I believe that it can be educational for a child without disabilities to play sports with my child

I struggle with organizing my schedule around my child's practices

Sports require a major economic investment

My child's sports allow me to have a greater social life

My child's participation in a sports activity has improved my life

Sports have allowed me to make meaningful relationships with other parents

I think it is important for my child to continue to participate in sports activities

Sports promote my child's autonomy

Sports help boost my child's self-esteem

Sports stimulate my child's socialization

Sports help my child acquire rules and discipline

I think sports help my child have and achieve goals

Sports help my child acquire new motor skills (e.g., coordination, attention, body pattern definition)

Sports allow my child not to perceive disability as a limitation

I am afraid that sports will expose my child to greater health risks (e.g., infections, fractures)

Sports improve my child's mental and physical health

# For my child, playing sports is primarily a/an *

## Mark only one oval.

Competitive activity

Activity of self-expression

Activity of fun and pleasure (playful)

Rehabilitative activity

1. How much do you think the following factors facilitated your child's ability to play sports?

*Mark only one oval per line.*

Not at all Little Fairly Much Very much

Knowing about leisure and sports activities suited to my child's needs

The attention of institutions that guarantee the right to sports

The presence of associations, third sector entities, cooperatives

The presence of competent and professional adults

Physical environment adaptation

The availability of appropriate communication tools

1. How much do you think the following elements are barriers for your child in practicing sports?

*Mark only one oval per line.*

Not at all Little Fairly Much Very much

Transportation

Unsuitable environments for complex situations

Architectural barriers

Cultural barriers (e.g., attitudes, behaviors, prejudices, beliefs, etc.)

High costs for sports practice

Absence of support for sports practice (e.g., wheelchair, prosthesis, etc.)

Lack of communication tools

# How does watching your child play sports make you feel? *

1. What would you say to a family who is on the fence about having their child with a disability start a sports activity?
