# Supplementary material for: A pilot study on sports activities in pediatric palliative care: just do it
Source: BMC Palliat Care. 2023 Apr 19;22:45. doi: 10.1186/s12904-023-01164-x (PMC10114486; doi:10.1186/s12904-023-01164-x)
Supplement: Supplementary file 3 — Supplementary material: Title of data: Supplementary Tables 1, 2, 3, 4, 5, 6. Description of data: Sports class characteristics, Equipment for sport, Barriers to sports activities for children in PPC, Thematic analyses. [file 12904_2023_1164_MOESM3_ESM.docx]

**Sports activities in pediatric palliative care: just do it**

**Supplementary Tables**

**Supplementary Table 1**. Sports class characteristics (source: original)

| ***Age*** | ***N=16*** |
| --- | --- |
| All the same age | 3 (19%) |
| Mixed age | 13 (81%) |
| ***Disability*** |  |
| Only people with disability | 12 (75%) |
| No other with a disability | 4 (25%) |
| ***Gender*** |  |
| Male | 1 (6%) |
| Female | 15 (94%) |

**Supplementary Table 2**. Equipment for sport.

| ***Equipment for sport*** | | | ***N=16*** | |
| --- | --- | --- | --- | --- |
| No equipment needed | | | 9 (56.3%) | |
| Equipment paid by the healthcare system | | | 1 (6.3%) | |
| Families who bought equipment | | | 2 (12.5%) | |
| Donated equipment | | | 2 (12.5%) | |
| Other | | | 2 (12.5%) | |
| **Supplementary Table 3.** Thematic analysis based on the open question for the caregiver: "How does watching your child play sports make you feel?" (Source: original) | | |  |  |
| **Thematic area** | **Verbatim quotes** | |  |  |
| Satisfaction | - Satisfied because I see him content - Satisfied by seeing him happy | |  |  |
| Happiness | - Happy - Good! - I am happy - Lighthearted - Wonderful… Happy that he enjoys and relates to everyone. - Fills me with joy - Serene | |  |  |
| Pride | - Proud - Proud - I feel proud of her. - Proud. - Enthusiastic and proud - She makes me feel good and proud of the achievements she can make - Immensely proud | |  |  |
| Equality | - An equal parent to others who have able-bodied children | |  |  |

| **Supplementary Table 4.** Thematic analysis based on the open question for the caregiver: “What would you say to a family who is on the fence about having their child with a disability start a sports activity?” (Source: original) | |
| --- | --- |
| **Thematic area** | **Verbatim quotes** |
| Encouragement | - I encourage her to try because it is a good opportunity - I encourage her to try it because it is worthwhile and satisfying - Absolutely to give it a try - That even with disabilities, great goals can be achieved - Encourage the family - There are already physical limitations; let’s not put limitations in our heads, too, in life we must always try; we can do anything as long as we do it our way, and if the way is not there, we invent it - I would tell them to try because the feelings are great - I would tell this family that anything is possible and limits are meant to be crossed - Try it to believe!!! - Let them do it because it will change their children's lives! |
| Socialization | - I would tell him to play sports to keep fit and make friends. - Absolutely do the sports activity most suitable for their child... sports integrate, include, give and bring happiness, enthusiasm, and fun |
| Equality and determination to achieve their own goals | - To let him feel "normal" - While doing apnea, I see my daughter competitive and happy with every accomplishment |
| Physical and psychological well-being | - Playing sports makes our children happy - I would focus on the following points: 1. overall well-being of the boy/girl, 2. Emancipation, 3. socialization, then the perception of a better quality of life - It is important for the psychological aspect of the child - That fears are our own and sports are good for everyone! |

| **Supplementary Table 5.** Thematic analysis based on the open question for the patient: “What do you like most about playing sports?” (Source: original) | |
| --- | --- |
| **Thematic area** | **Verbatim quotes** |
| Sociality | - Teamwork - Being in company - Being with others - Being with others - Playing and being with other people - Being around people - Meeting new friends |
| Feeling of freedom | - I have fun and go at high speed without being called back - Having fun - Reading, singing, and playing music - Somersaulting in the water - Playing - What I want to do - Being able to move without ever falling |
| Competition | - Putting myself in competition |
| Nothing | - Nothing |

| **Supplementary Table 6.** Thematic analysis based on the open question for the patient: “What would you say to a child with disabilities who is unsure whether to start a sports activity? (Source: original) | |
| --- | --- |
| **Thematic area** | **Verbatim quotes** |
| Encouragement | - To try it, he will find that he enjoys it - That they should try it because it is a way to have fun together and take their mind off various thoughts or problems - To follow one's dreams - Do it - To try it - To try it - Try it, maybe you will discover a passion and a new way of considering yourself. If you don't try, nothing changes - To start to try - I would tell them that nothing is impossible: try and see - To start immediately!!! That is the greatest thing ever! |
| Physical and psychological well-being | - You have to play sports because it is good for you and because you make many friends - To start because it is fun - To do it because it is much fun - I would tell them to start playing a sport because it's good for them and keeps them fit - To have fun together - I would tell them to start a sport because it changes their life |

**Supplementary Table 7.** Barriers to sports activities for children in PPC.

| ***Statement*** | ***Not at all*** | ***Little*** | ***Sufficiently*** | ***Much*** | ***Very much*** |
| --- | --- | --- | --- | --- | --- |
| Transportation | 6  (38%) | 2  (13%) | 4  (25%) | 2  (13%) | 2  (13%) |
| Unsuitable environments | 3  (19%) | 4  (25%) | 1  (6%) | 8  (50%) | – |
| Architectural barriers | 4  (25%) | 2  (13%) | – | 5  (31%) | 5  (31%) |
| Cultural barriers | 3  (19%) | 2  (13%) | 2  (13%) | 6  (38%) | 3  (19%) |
| High costs | 3  (19%) | 5  (31%) | 1  (6%) | 3  (19%) | 4  (25%) |
| Lack of support | 5  (31%) | 2  (13%) | 1  (6%) | 3  (19%) | 5  (31%) |
| Lack of communication tools | 6  (38% | 4  (25%) | 2  (13%) | 4  (25%) | – |
